# A survey on the attitudes towards research in medical school

**DOI:** 10.1186/1472-6920-10-4

**Published:** 2010-01-22

**Authors:** D Robert Siemens, Sanoj Punnen, James Wong, Nimira Kanji

**Affiliations:** 1 Department of Urology and the Centre for Applied Urological Research, Queen's University, Kingston, Ontario, Canada

## Abstract

**Background:**

An observed decrease of physician scientists in medical practice has generated much recent interest in increasing the exposure of research programs in medical school. The aim of this study was to review the experience and attitudes regarding research by medical students in Canada.

**Methods:**

An anonymous, cross-sectional, self-report questionnaire was administered to second and fourth year students in three medical schools in Ontario between February and May of 2005. Questions were primarily closed-ended and consisted of Likert scales. Descriptive and correlative statistics were used to analyze the responses between students of different years and previous research experience.

**Results:**

There was a 47% (327/699) overall response rate to the questionnaire. Despite 87% of respondents reporting that they had been involved in some degree of research prior to medical school, 43% report that they have not been significantly involved in research activity during medical school and 24% had no interest in any participation. There were significant differences in the attitudes towards research endeavors during medical school between students in their fourth year compared to second year. The greatest barriers to involvement in research in medical school appear to be time, availability of research mentors, formal teaching of research methodology and the perception that the student would not receive appropriate acknowledgement for work put towards a research project.

**Conclusion:**

The results of this self-report survey outline the significant differences in attitudes towards mandatory research as a component of critical inquiry and scholarship in the undergraduate curriculum in Ontario medical schools.

## Background

There has been a documented decline in the number of physician- scientists in medical practice [1]. A number of technical-based specialties have expressed concern of professional stagnation without the constant reconstruction afforded by the development of novel clinical and basic science knowledge [2-4]. Postulated explanations for the decline of the physician-scientist include less financial incentive, family, practice philosophy and inadequate exposure to research before career paths are determined [5,6]. The Royal College of Physicians and Surgeons of Canada has adopted the Scholar role as one of the core competencies of specialty training that has translated to the expectation of a contribution to research in training. However, published studies regarding resident and medical student research in specialty training has documented ambivalent attitudes regarding its value [2,7-14] with as much as 75% of residents preferring to engage in other scholarly activities as compared to research [2]. Other proposed remedies to reverse this disinclination towards basic science or clinical research output by practicing physicians include the implementation of MD-PhD programs, fostering research in sub-specialty fellowships and increased exposure to research at the medical school level by means of medical scientist training programs [1,15-18]. Furthermore, there has been a significant movement towards providing medical students with early research experience within the medical school curriculum [5,16,17].

However, given the demands and competing interests of formulating an undergraduate medical curriculum, as well as the results of attitudes of other learners during medical training, it appears pivotal to inquire into the experience of research during medical school. Our objectives of this study were to survey Canadian medical students' experience and attitudes towards research and investigate their perceived goals and barriers to such endeavors during their educational experience.

## Methods

This prospective study surveyed medical students from three medical schools (Queens University, University of Ottawa and University of Western Ontario) within Ontario. Students from both the second and fourth year classes from all three schools during the 2005 academic year were invited to participate in this survey, although students enrolled in joint MD-PhD programs were excluded. Participation within these representative classes was completely voluntary and confidentiality was maintained at all times as no identifying information was recorded in the survey results. Students were contacted between class lectures or teaching sessions and invited to participate in the survey by study representatives (S.P., J.W., N.K) who were also students in the respective medical school at the time. Ethics approval was attained from each institutional review board and explanations for the objectives of the study and assurance of confidentiality was distributed to the students responding to the survey.

The questionnaire consisted of 33 closed-ended questions addressed to report the experience and attitudes of Canadian medical students to explore a number of issues: why do students choose to be involved in research; what is the relevance of student research to career aspirations; what are the barriers to successful participation in research; are students adequately exposed to research methodology and critical appraisal? The survey was made available in both English and French and took on average only five to ten minutes to complete. The first 11 questions of the survey assessed demographic information, research background and career aspirations for the students being surveyed. The remaining 22 questions were in Likert scale format and addressed the above-mentioned objectives. Questionnaire development resulted from an initial experience with a previous survey construction for similar attitudes for specialty residents. Students and educators involved in both under-graduate and post-graduate programs were asked to assess and modify the survey for clarity. The second year and fourth year students were felt to be a good representation of the range of medical school experience and were used to compare any changing attitudes throughout time.

Descriptive statistics were used to describe demographics and research background of students. For ease of reporting, descriptive differences using the agreement responses of 4 and 5 to were grouped together, as were the disagreement responses of 1 and 2. All other quantitative statistics utilized the full 5-point Likert scale. Bivariate analysis with [chi]^2 ^or a Kruskal-Wallis test was used to determine statistical differences between demographic data and research experience between schools, second and fourth year students and those with and without graduate degrees. A Mann-Whitney test was used to compare Likert scale scores between respondents with and without previous research experience as well as between years of education. Spearman or Pearson tests, depending on normality of distribution, were used to demonstrate correlations of respondents to questions using the Likert scale. The StatView^® ^statistical software package (Abacus Concepts, Inc., Berkley, CA) was used for analysis.

## Results

The overall response rate of the two classes from the three medical schools given the survey was 47% (327/699). The overall response rates between medical schools were similar with 113/190 (54%) from Queen's University, 98/250 (39%) from University of Western Ontario and 116/259 (45%) from the University of Ottawa. The response rate from the second year classes (185/368; 50%) was similar to that of the fourth year classes (142/331; 43%); however, the response rates between the second and fourth year class did vary between the different medical school programs: University of Ottawa (78% vs. 18% respectively), Queen's University (45% vs. 76%), University of Western Ontario (35% vs. 44%). There was minimal variation of demographic data and background information regarding research experience between respondents from the classes of the different medical schools and these results were similar to a recently published survey on the characteristics of medical students in other Canadian medical schools [19].

The mean age +/- standard deviation of the respondents from Queen's was (26.3 +/- 2.5), University of Western Ontario (25.1 +/- 1.9) and University of Ottawa (26.0 +/- 2.8). There was no significant difference in mean age between schools in each of the two medical school class years. Stratifying between schools, there was no difference of respondents who reported no or minimal research experience before attending medical school (Queen's University 16%; University of Western Ontario 13%; University of Ottawa 9%) although there was significantly smaller proportion of respondents reporting completion of graduate training, including a Master's or PhD. (University of Western Ontario 7%; Queen's University 28%; University of Ottawa 28%) (p = 0.01, chi^2 ^test). The percentage of respondents who reported a primary interest in family medicine as a career (compared to Royal College specialty programs) did not differ between the three schools (Queen's University 24%; University of Western Ontario 27%; University of Ottawa 25%). When asked to evaluate the perceived competitiveness of the respondents desired post-graduate training program, and therefore a possible function of research involvement, answers did not vary between different medical schools (p = 0.9730, Kruskal-Wallis test). Given this homogenous demographic background, it was decided to combine responses of students from the three Ontario medical schools and focus comparisons on differences of second and fourth year students as well as those with and without graduate degrees.

Demographic data and level of past and present research experience for the respondents categorized by year of medical training are represented in Tables 1. There was no significant difference between second and fourth year students in their previous research experience before medical school and it appeared that there was no difference in the perceived competitiveness of their desired residency choice. It was apparent however that the involvement in research activities increases greatly between second and fourth year with 49% of second year students responding that they had little or no participation in research compared to only 14% of fourth year students. Despite the majority of fourth year medical students involving themselves in research to some degree, only 36% responded positively that they would anticipate a good understanding of research methodology by the time they graduate. The type of research reported by the students was generally involvement in retrospective chart reviews or case reports with only 9% reporting involvement with basic science projects.

**Table 1:** Comparison of demographic data and research experience in medical school for respondents in second and fourth year medical school

	2nd Year Students N = 185	4^th ^Year Students N = 142	P value for trend
Age (SD)	25.2 (1.28)	26.7 (1.8)	<0.001
Graduate degrees	24%	18%	0.22
Primary interest in family medicine	27%	28%	0.80
Never presented research in medical school	58%	30%	0.001
Significant involvement in research projects	19%	29%	0.0002
Interested in a "competitive" residency	43%	47%	0.68
No involvement in research projects	49%	14%	0.0004
Anticipate a good understanding of research methodology	23%	36%	0.10

As anticipated, there were striking differences in the respondents with and without graduate degrees regarding research experience during medical school (Table 2). Those with previous graduate schoolwork appeared to have less interest in family practice as a preferred career choice although there was a similar perception of the competitiveness of their primary residency position compared to those without a graduate degree. Those with a graduate degree reported higher likelihood to be actively involved in research activities in medical school (43% vs. 16%), publishing research papers (47% vs. 21%) and anticipate a good understanding of research methodology after their education (64% vs. 18%).

**Table 2:** Comparison of demographic data and research experience in medical school for respondents with and without graduate degree work

	Graduate degree N = 72	No graduate degree N = 255	P value for trend
Age (SD)	27.8 (2.3)	25.4 (2.3)	<0.0001
Primary interest in family medicine	12%	29%	0.003
Never presented research in medical school	35%	48%	0.03
Published research paper	47%	21%	<0.0001
Significant involvement in research projects	43%	16%	<0.0001
Interested in a "competitive" residency	44%	45%	0.35
No current involvement in research projects	28%	35%	0.049
Anticipate a good understanding of research methodology	64%	18%	<0.0001

Within the survey, the medical students were asked about their attitudes towards their education in research methodology and their involvement in research projects, as well as perceived barriers to becoming involved in research during medical school. The vast majority of all of the responders (83%) agreed that some participation in research was likely valuable within their medical education; however, only 44% all responders agreed or strongly agreed that research will play a significant role in their future career and only 38% agreed or strongly agreed that more time in medical school should be set aside to allow participation in research endeavors. Forty three percent of respondents agreed that the main reason for their participation in research was to facilitate acceptance into their residency of choice. Time was seen to be a significant barrier to pursuing research as only 31% of respondents felt there was adequate time set aside. Furthermore, only 15% of respondents felt that there was sufficient training in research methodology in medical school and only 25% agreed that there was adequate training in critical appraisal of scientific literature. Another perceived barrier to participation in research was the difficulty in attaining a research supervisor with only 44% of respondents agreeing that it was relatively easy to find a research mentor. However, the majority of respondents reported a good level of satisfaction with their research mentors and only 14% felt that they would not get appropriate acknowledgement of their contributions to a research project.

The responses regarding attitudes towards research and the perceived barriers to research for those in second and fourth year as well as those with and without graduate degrees can be found in Tables 3, 4, 5 and 6. It is apparent from these responses that the level of involvement and the appreciation for research changes somewhat in the higher years of medical education. Perceived barriers to research participation, including the access to research mentors and available time, still appears significant for the fourth year class, although somewhat less of an obstacle compared to the second year class. As could be predicted, positive attitudes towards research activities in medical school were apparent in those with previous graduate degree work.

**Table 3:** Comparison of attitudes regarding research interest for respondents in second and fourth year medical school

	Agreement* from 2nd Year Students N = 185	Agreement* from 4^th ^Year Students N = 142	P value for trend**
Involved in research to facilitate admission to residency	37%	51%	0.274
Research will be a part of long-term career goals	38%	53%	0.041
Involved in research because of interest in the field	48%	65%	0.033
No interest in research	27%	18%	0.026
Research is not relevant to medical education	17%	18%	0.665
Research should not be an important criteria for acceptance to residency	66%	55%	0.008
Mandatory research time should be set in medical school curriculum	34%	46%	0.328

*Responses 4 and 5 in 5-point Likert scale were grouped as "agreement" for reporting purposes.

**Mann-Whitney test between responses of 5-point Likert scale

**Table 4:** Comparison of attitudes regarding barriers of research for respondents in second and fourth year medical school

	Agreement* from 2nd Year Students N = 185	Agreement * from 4^th ^Year Students N = 142	P value for trend**
Adequate time in medical school to pursue research	16%	25%	0.0059
Adequate training in research methodology in medical school	12%	9%	0.901
Adequate training in reviewing scientific literature	25%	32%	0.292
Research mentors are easily available	36%	52%	0.0325
Research supervisors offer good training and guidance	22%	37%	0.180
Many opportunities to present research in medical school	16%	20%	0.246
Many opportunities to publish research during medical school	15%	25%	0.397
Will receive acknowledgment for contributions to research	30%	58%	0.002

*Responses 4 and 5 in 5-point Likert scale were grouped as "agreement" for reporting purposes.

**Mann-Whitney test between responses of 5-point Likert scale

**Table 5:** Comparison of attitudes regarding research interest for respondents with and without graduate degree work

	Agreement* from those with graduate degree N = 72	Agreement* from those without graduate degree N = 255	P value for trend**
Involved in research to facilitate admission to residency	46%	42%	0.643
Research will be a part of long-term career goals	55%	42%	0.016
Involved in research because of interest in the field	65%	52%	0.048
No interest in research	10%	28%	<0.0001
Research is not relevant to medical education	15%	18%	0.0234
Research should not be an important criteria for acceptance to residency	47%	65%	0.007
Mandatory research time should be set in medical school curriculum	46%	37%	0.109

*Responses 4 and 5 in 5-point Likert scale were grouped as "agreement" for reporting purposes.

**Mann-Whitney test between responses of 5-point Likert scale

**Table 6:** Comparison of attitudes regarding barriers of research for respondents with and without graduate degree work

	Agreement from those with graduate degree N = 72	Agreement from those without graduate degree N = 255	P value for trend**
Adequate time in medical school to pursue research	19%	20%	0.327
Adequate training in research methodology in medical school	8%	12%	0.214
Adequate training in reviewing scientific literature	26%	29%	0.550
Research mentors are easily available	47%	42%	0.289
Research supervisors offer good training and guidance	42%	24%	0.0027
Many opportunities to present research in medical school	24%	16%	0.307
Many opportunities to publish research during medical school	13%	20%	0.115
Will receive acknowledgment for contributions to research	58%	38%	0.0009

*Responses 4 and 5 in 5-point Likert scale were grouped as "agreement" for reporting purposes.

**Mann-Whitney test between responses of 5-point Likert scale

In an attempt to better appreciate and understand the attitudes towards research reported in this survey, correlations were made of some of the responses by the fourth year medical students. There was a positive correlation with applicants' involvement in research and those responding that their preferred residency positions were perceived to be highly competitive (correlation coefficient 0.41, p-value < 0.001). Those who were involved in research to attain a preferred residency position were more likely to feel research was relevant to their long-term career goals (correlation coefficient 0.568, p < 0.001) and that there was sufficient time set aside in medical school to participate in research (correlation coefficient 0.282, p = 0.022). Furthermore, those students who felt it was easy to identify a research mentor were more likely to be interested in research during medical school (correlation coefficient 0.236, p = 0.05), feel that they had a good training in critical appraisal (correlation coefficient 0.254, p = 0.03) and express a future interest in research as a career goal (correlation coefficient 0.23, p = value 0.015).

## Discussion

We have found great disparity regarding the participation in and attitudes towards research by Canadian medical students, as well as significant barriers impeding these activities during their education. Although the majority of those medical students responding to this survey felt that participation in research activities was likely beneficial to their education, only 44% felt that research will play a significant role in their future career and only 38% agreed that more time should be set aside in medical school to facilitate more research experience.

The rationale for facilitating research experience during a medical school education includes the development of an appreciation for research methodology, and subsequent critical appraisal of the medical literature, as well as to foster the interest in medical or basic science research as an academic career [5,16,17]. There are numerous conflicting priorities in any medical school curriculum and the role of any mandatory research project or critical inquiry must be balanced against other demands of knowledge and skill acquisition. Despite the documented concern regarding the decline of clinician-scientists in North America [1,2,7-10], as well as published strategies suggested to reverse these trends at the level of medical school training [1,15-18], the experience and attitudes towards research of students enrolled in medical schools has not been recently examined.

The results from the present study underscore the variable experience and attitudes towards research during medical school and are consistent with similar studies of those students in residency programs. Of those students responding to the survey, 43% stated that they had no significant involvement in research projects during medical school and 24% had no interest in any research endeavors. Forty three percent of respondents agreed that the main reason for participation in research during medical school was to facilitate acceptance into a residency of choice.

There are numerous barriers to research participation during the education and training of a physician including time commitments within and outside of medicine [5,7]. Time was seen to be a significant barrier to pursuing research during medical school as only 31% of all respondents felt there was adequate allotted time for research endeavors. Furthermore, only 15% of respondents felt that there was sufficient training in research methodology in medical school and only 25% agreed that there was adequate training in critical appraisal of scientific literature. Another perceived barrier to participation in research was the difficulty in attaining a research supervisor with only 44% of respondents agreeing that it was relatively easy to find a research mentor. Comparing the responses of second and fourth year students it was apparent that some of these barriers were less constraining during the later years of their education, including perceived available time and access to research mentors. This is likely due to more access to clinical rotations in later years of medical school, facilitating opportunities for research endeavors. Although the interest and participation in research was more apparent for those students with previous graduate work there was little difference in their perceived limitations of time and opportunity compared to those without graduate degrees.

There are several limitations to this study that need to be considered. First, the results are derived from a self-report survey on the participation and attitudes towards research in medical school and independent verification of data was not possible. Secondly, some respondents chose not to answer all questions in the survey; however we feel that this effect is negligible as the question most often left unanswered was regarding ongoing interest in research, and it was unanswered by only 5% of the students. This study represents the research experience and attitudes of only three Canadian medical schools, all in Ontario, and may not be representative of those from other areas. Finally, the response rate of 47% is somewhat less than what was expected and this is likely related to the decision to approach the student body with a paper copy of the study rather than an electronic version. It was the experience of the volunteers that those approached to complete the survey did so, however, the attendance of the students at the targeted classes or seminars was not complete. We feel that this response rate is therefore likely a good representation of the experience and attitudes towards research in medical school and is acceptable for this type of study and similar to others [8,11,12].

The data we present are relevant to discussions regarding research within a medical school curriculum in a number of areas. Although the vast majority of respondents acknowledged the importance of understanding research methodology in their education as physicians, there appears to be diversity in opinion regarding the institution of mandatory research projects within medical school. Consistent with opinion from reports on research during residency programs, these results suggest that it is unlikely that any one strategy to educate future physicians on the principles of research, such as mandatory research projects, would suffice to meet every student's particular needs and interest. If there is a desire to advance the interest and participation of research within a medical school curriculum, it may be beneficial to facilitate more opportunity and direction in the earlier years of education. It also seems apparent that there are students (i.e. those with graduate degree work) who could be identified early as more likely to be interested in participation in medical research. Finally, beyond the lack of available time, there appears to be other significant barriers to the participation in research during medical school including the availability of research mentors as well as a perceived lack of formal education in research methodology and critical appraisal.

## Conclusion

The results of this self-report survey outline the significant differences in attitudes towards mandatory research as a component of critical inquiry and scholarship in the undergraduate curriculum in Ontario medical schools.

Future work should include a wider survey of these pertinent questions regarding the role of research in medical school. Furthermore, a similar survey of other stakeholders in medical education, including educators in the undergraduate and post-graduate programs, would be highly informative, especially regarding the role and perceived barriers to research in the medical school curriculum.

## Competing interests

The authors declare that they have no competing interests.

## Authors' contributions

D.R.S. conceived and designed the study and drafted the final manuscript. At the time of the survey S.J. was a medical student at Queen's University, conceived of the study, participated in disseminating and collecting the survey and helped in drafting the manuscript. J.W. was a medical student at the University of Ottawa, participated in development, dissemination and collection of the survey and helped in drafting the manuscript. N.K. was a medical student at the University of Western Ontario and participated in the dissemination and collection of the survey. All authors's read and approved of the final manuscript.

## Pre-publication history

The pre-publication history for this paper can be accessed here:

http://www.biomedcentral.com/1472-6920/10/4/prepub
